# A Machine Learning-Based System for Real-Time Polyp Detection (DeFrame): A Retrospective Study

**DOI:** 10.3389/fmed.2022.852553

**Published:** 2022-05-31

**Authors:** Shuijiao Chen, Shuang Lu, Yingxin Tang, Dechun Wang, Xinzi Sun, Jun Yi, Benyuan Liu, Yu Cao, Yongheng Chen, Xiaowei Liu

**Affiliations:** ^1^Department of Gastroenterology, Xiangya Hospital of Central South University, Changsha, China; ^2^National Clinical Research Center for Geriatric Disorders, Xiangya Hospital Central South University, Changsha, China; ^3^Hunan International Scientific and Technological Cooperation Base of Artificial Intelligence Computer Aided Diagnosis and Treatment for Digestive Disease, Hunan, China; ^4^HighWise Medical Technology Co., Ltd., Suzhou, China; ^5^Department of Computer Science, The University of Massachusetts Lowell, Lowell, MA, United States; ^6^Department of Oncology, NHC Key Laboratory of Cancer Proteomics & State Local Joint Engineering Laboratory for Anticancer Drugs, National Clinical Research Center for Geriatric Disorders, Xiangya Hospital, Central South University, Changsha, China

**Keywords:** artificial intelligence, convolutional neural networks, deep learning, colonoscopy, computer-aided detection

## Abstract

**Background and Aims:**

Recent studies have shown that artificial intelligence-based computer-aided detection systems possess great potential in reducing the heterogeneous performance of doctors during endoscopy. However, most existing studies are based on high-quality static images available in open-source databases with relatively small data volumes, and, hence, are not applicable for routine clinical practice. This research aims to integrate multiple deep learning algorithms and develop a system (DeFrame) that can be used to accurately detect intestinal polyps in real time during clinical endoscopy.

**Methods:**

A total of 681 colonoscopy videos were collected for retrospective analysis at Xiangya Hospital of Central South University from June 2019 to June 2020. To train the machine learning (ML)-based system, 6,833 images were extracted from 48 collected videos, and 1,544 images were collected from public datasets. The DeFrame system was further validated with two datasets, consisting of 24,486 images extracted from 176 collected videos and 12,283 images extracted from 259 collected videos. The remaining 198 collected full-length videos were used for the final test of the system. The measurement metrics were sensitivity and specificity in validation dataset 1, precision, recall and F1 score in validation dataset 2, and the overall performance when tested in the complete video perspective.

**Results:**

A sensitivity and specificity of 79.54 and 95.83%, respectively, was obtained for the DeFrame system for detecting intestinal polyps. The recall and precision of the system for polyp detection were determined to be 95.43 and 92.12%, respectively. When tested using full colonoscopy videos, the system achieved a recall of 100% and precision of 80.80%.

**Conclusion:**

We have developed a fast, accurate, and reliable DeFrame system for detecting polyps, which, to some extent, is feasible for use in routine clinical practice.

## Introduction

Colorectal cancer (CRC) is one of the most common malignancies and the fourth leading cause of cancer-related mortality worldwide (1, 2). Adenomatous polyps, precancerous lesions, will eventually progress to CRC without proper timely intervention. Colonoscopy, as a tool to effectively detect and resect polyps, has played an essential role in preventing the development of CRC. Therefore, it is considered to be the gold standard method for CRC screening (3).

However, some polyps are still missed during the gold standard procedure (4). This procedure involves various factors, such as the endoscopist’s experience, bowel preparation (5), intestinal mucosal exposure (6), and imaging equipment (7, 8). According to statistics, approximately one-fourth of adenomatous polyps can be missed in clinical practice (9), which is the main cause of interval CRC (10, 11). Several indicators have been established to improve the quality of colonoscopy and its performance, among which the adenoma detection rate (ADR) is well known as an independent indicator because of its negative correlation with CRC-related mortality (12–14).

In recent years, with the continuous development of artificial intelligence (AI), especially the emergence and use of deep learning and convolutional neural networks (CNNs) that can automatically extract features and recognize images, image recognition has led to constant breakthroughs, the level of which has gradually reached that of humans. Because of this feature, many researchers have been attracted to developing and optimizing AI-assisted polyp detection systems (15–19). However, most current studies are based on public and small- to medium-sized private datasets; the data volume is not large enough to perform adequate AI deep learning. On the other hand, most of these studies use only one kind of deep learning algorithm, which has not been validated in a clinical setting. Additionally, most of these studies utilize only traditional parameters - specificity and sensitivity–to assess the system’s performance. In our opinion, other novel indicators should also be included to evaluate performance to gain a deeper understanding of a system’s contribution in a clinical setting. In this study, we first developed a machine learning (ML)-based polyp detection system, named DeFrame. Compared with other existing polyp detection systems, the DeFrame system integrates two deep learning (DL)-based algorithms for polyp detection and a fuzzy image filtering module to obtain a more accurate output. In addition, to mock its application in a clinical setting, we tested the DeFrame system with full videos generated during routine endoscopy. The results obtained are quite good. To our knowledge, the database we used to develop the DeFrame system is the largest thus far, which combines multiple open-source datasets and self-built image and video datasets. Finally, we carefully evaluated the system’s performance with both image and target measurement parameters.

## Materials and Methods

### Medical Records

We retrospectively collected full colonoscopy videos for 824 patients who received a colonoscopy between June 2019 and June 2020 in the endoscopy rooms of the Gastroenterology Department, Xiangya Hospital, Central South University. According to the patient inclusion and exclusion criteria, 681 videos were included for further studies. A group of experienced endoscopists (with at least 2 years of experience) from Xiangya Hospital identified all the polyps on the videos and annotated the time of appearance and disappearance and the size and location of each polyp. If a polyp was removed during the colonoscopy and sent for histology, the corresponding pathology report was collected and reviewed for accurate diagnosis by another pathologist from Xiangya Hospital. A video clip containing the polyp from the time of its appearance to the time of its disappearance was then cut and saved to extract images containing diversified artifacts and polyps.

### Inclusion and Exclusion Criteria for the Participants

The inclusion criteria were adults aged 18 years and older undergoing a nonemergent colonoscopy.

The exclusion criteria were as follows: (1) familial multiple polyposis or inflammatory bowel disease; (2) contraindications to biopsy; (3) personal history of CRC or CRC-related surgery; and (4) presence of submucosal lesions.

### Main Equipment

All colonoscopies were performed using a high-definition endoscope (Olympus CV70/CV260) and recorded using high-definition recorders (HDTVs). All the collected videos were taken in white light, noniodine staining, and nonmagnification mode.

### The Development of the DeFrame Polyp Detection System

**FIGURE 1:**
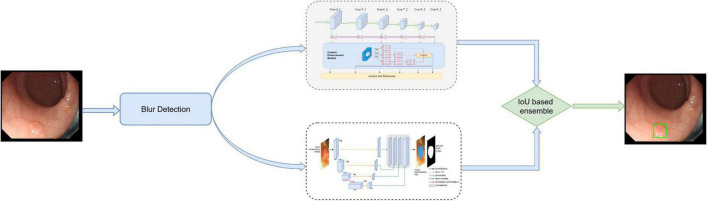
The architecture of our developed system, which consists of blurry detection, polyp detection, and fusion module. Among them, polyp detection was performed by two algorithms (algorithm B based on AFP-Net and algorithm C based on U-Net). Data flow is from the left to the right: images were first detected by blurry detection module (algorithm A) and transferred to polyp detection module if they were clear. Output was then gained with a bounding box on the CADe monitor.

The initial DeFrame system was constructed using images obtained from public databases. This system integrates three algorithms, including Algorithms A, B, and C, and a fusion component. Algorithm A is used to determine whether the image is blurred (20). Both Algorithms B (based on AFP-Net) (21) and C (based on dilated U-Net) (22) are polyp detection modules. The primary work process is described as follows: single-frame images from colonoscopy are used as inputs; the blurred images are filtered out by Algorithm A; polyps are detected by Algorithms B and C simultaneously. The specific process is shown in Figure 1. For the training details, Algorithm C was trained on 2 Nvidia Geforce 1080ti’s with an initial learning rate of 1e^–5^, while Algorithm B was trained on 4 Nvidia RTX 2080ti’s with an initial learning rate of 1e^–3^.

### The Datasets for Training and Validation

The training datasets consisted of 1,544 images from public databases and 6,833 images extracted from self-collected videos. All images from the public polyp datasets (CVC-Clinic DB (23), ETIS-Larib polyp DB (24), GIANA Polyp Detection/Segmentation (25), and CVC-Colon DB (26)) have at least one polyp. The images were extracted from 48 videos we collected from June 2019 to July 2019, and 81.00% of the images contained at least one polyp. Validation set 1 included 24,486 images extracted from 176 videos collected between July 2019 and September 2019, and 42.60% of images contained at least one polyp. Validation set 2 included 12,283 images extracted from 259 videos collected between September 2019 and December 2019, and 84.90% of the images contained at least one polyp. Validation set 2 was built to validate the system’s ability for polyp localization and accurate polyp image segmentation. Validation set 3 included 198 full videos collected from December 2019 to January 2020, serving as the test set. A total of 344 polyps were found in validation set 3. The specific composition of each dataset is shown in Table 1.

**TABLE 1:** Summary of datasets used for model training and validation.

	Development dataset		Validation dataset		
Characteristics	Self-built subset	Public subset	Dataset 1	Dataset 2	Dataset 3 (video)
Images	6833	1544	24,486	12,283	.
Images with polyp	5513	1544	10,424	10,424	.
Images without polyp	1320	0	14,062	1,859	.
Polyps	223	170	540	140	344
**Pathology**					
Hyperplastic, n (%)	22 (9.87)	NA	115 (21.31)	19 (13.57)	8 (2.33)
Inflammatory, n (%)	30 (13.45)	NA	99 (18.41)	28 (20.00)	165 (47.97)
Adenoma, n (%)	131 (58.74)	NA	260 (48.07)	77 (55.00)	117 (34.01)
Carcinoma, n (%)	38 (17.04)	NA	58 (10.74)	11 (7.86)	27 (7.85)
Others	2 (0.90%)	NA	8 (1.48)	5 (3.57)	27 (7.85)
**Polyp location**					
Cecum, n (%)	22 (11.22)	NA	11 (2.09)	3 (2.14)	23 (7.80)
Ascending, n (%)	83 (42.35)	NA	35 (6.52)	12 (8.57)	55 (18.64)
Transverse, n (%)	20 (10.20)	NA	75 (13.96)	22 (15.71)	49 (16.61)
Descending, n (%)	24 (12.24)	NA	200 (37.01)	50 (35.71)	71 (24.07)
Sigmoid, n (%)	31 (15.82)	NA	50 (9.34)	12 (8.57)	53 (17.97)
Rectum, n (%)	16 (8.16)	NA	83 (15.29)	41 (29.29)	44 (14.91)
**Polyp size/shape**					
Small (≤5 mm)	118 (60.20)	NA	115 (21.31)	47 (33.57)	134 (38.95)
Isochromatic, n (%)	104 (53.06)	NA	99 (18.41)	37 (26.43)	37 (10.76)
Flat, n (%)	94 (42.03)	NA	260 (48.07)	43 (30.71)	79 (22.97)

*NA, Not applicable. Public dataset does not provide the detailed polyp information. Dataset 3 is in the form of unaltered videos, so no images were available.*

### Outcome Evaluation Metrics

In this study, we evaluated the performance of the DeFrame system in terms of both images and polyps. The former includes the following metrics: sensitivity and specificity, the results of which were demonstrated in validation set 1; the latter includes recall, precision, and F1 score, the results of which were demonstrated in validation set 2. The overall performance was assessed from the video perspective with validation set 3.

### Statistical Analysis

The sample size for this study was sufficient to ensure that the results we obtained were adequate to produce statistically significant differences. The sample size calculation process is described as follows: first, the typical sample size calculation formula [e.g., ss = ($s\,s=\frac{z_{1-\alpha /2}^{2}*\left(p\right)*\left(1-p\right)}{C}$] was used to calculate the initial sample size with a preset *z*-value of 1.96 for the confidence level, α of 0.05, p of 0.90, and c of 0.01. Therefore, we found that the sample size should be at least 3,457. To further refine the sample size, we also researched recent papers on the development and validation of deep learning-based polyp detection systems. We found that the number of images they used ranged from 8,000 to 30,000. Therefore, we decided to set the sample size to 24,486 and 12,283 for validation datasets 1 and 2, respectively.

Python scripts were used to calculate the evaluation metrics in this study.

## Results

### Demographic Information

**FIGURE 2:**
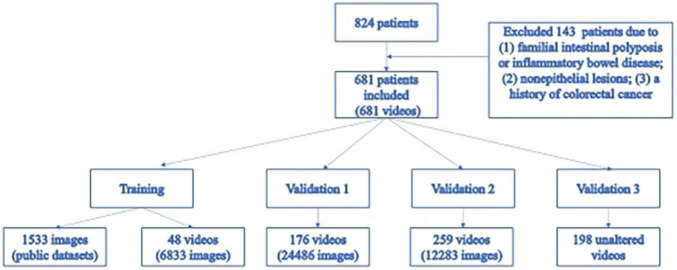
Flowchart of the research.

A total of 681 eligible subjects were enrolled, among which 463 were male (68.00%). The median age was 65 years, and the interquartile range (IQR) was 56–75. A video recording of the entire colonoscopy was obtained for each subject, and in total, 1,240 polyps were found in 681 videos (Figure 2).

### Differences in the Working Performance of Our Developed Polyp Detection Algorithm and Other Current Algorithms

When trained and tested in two public polyp datasets (CVC-Clinic DB and ETIS-Larib Polyp DB), the algorithms (including B and C) and DeFrame system we developed outperformed all other algorithms reported in the literature for polyp detection. The specific values for each algorithm are shown in Table 2.

**TABLE 2:** Performance comparison of different polyp detection algorithms and systems on two public datasets.

	CVC-ClinicDB			ETIS-Larib Polyp DB		
Algorithms/Systems	Precision (%)	Recall (%)	F-1 score	Precision (%)	Recall (%)	F-1 score
CVC-Clinic	83.50	83.10	83.30	10.00	49.00	16.50
ASU	97.20	85.20	90.80	NA	NA	NA
OUS	90.40	94.40	92.30	69.70	63.00	66.10
CUMED	91.70	98.70	95.00	72.30	69.20	70.70
Faster R-CNN	86.60	98.50	92.20	NA	NA	NA
FCN	89.99	77.32	83.00	NA	NA	NA
FCN-8S	91.80	97.10	94.38	NA	NA	NA
FCN-VGG	NA	NA	NA	73.61	86.31	79.46
Algorithm B	99.36	96.44	97.88	88.89	80.77	84.63
Algorithm C	96.71	95.51	96.11	80.48	81.25	80.86
DeFrame system	98.85	92.88	95.77	91.02	73.08	81.07

*The last three rows show the results of our proposed algorithms (Algorithm B and C) and the DeFrame system. Other rows show the results from existing methods. NA, Not applicable.*

### Image Classification Results for the DeFrame System

**FIGURE 3:**
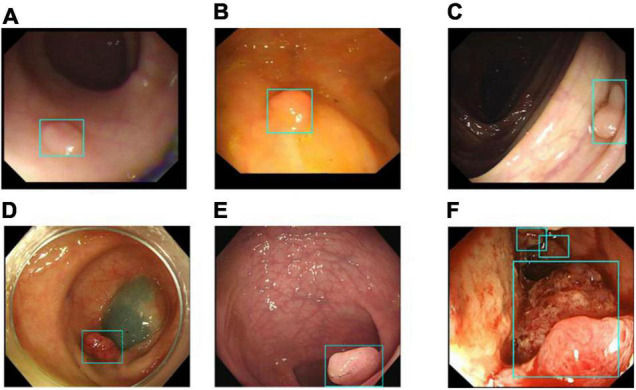
Images **(A–F)** showing that the DeFrame system generates the correct output, regardless of location and morphology. Specifically, light-blue boxes are used to indicate where the polyps are detected. **(A)** A small and flat hyperplastic polyp in the sigmoid colon; **(B)** an isochromatic and small inflammatory polyp in the sigmoid colon; **(C)** an adenomatous polyp in the ascending colon, **(D)** an adenomatous polyp in the descending colon, **(E)** an adenomatous polyp in the rectum; **(F)** multiple carcinomatous polyps in the sigmoid colon.

Image classification refers to the DeFrame system’s ability to automatically distinguish whether the image contains polyps. The results of colonoscopy image classification in validation set 1 are shown in Table 3. The sensitivity was 79.54%, and the specificity was 95.83%. In addition, the system can identify polyps with various histological features and sizes in different locations. The output results for the DeFrame system to correctly distinguish the images are shown in Figure 3.

**TABLE 3:** Image classification results of the DeFrame system.

	Dataset1
True positives	8,224
False negatives	2,200
True negatives	13,476
False positives	586
Sensitivity	79.54%
Specificity	95.83%

### Target Detection Results for the DeFrame System

We used validation set 2 to evaluate the DeFrame system performance in polyp localization and segmentation (i.e., identifying and segmenting every polyp in one image). The target detection results are shown in Table 4.

**TABLE 4:** Object detection results of the DeFrame system.

	Dataset 2
Target regions	10,586
Segmentation regions	10,966
Overlapped regions between target regions and segmentation regions	10,102
Recall	95.43%
Precision	92.12%
F-1 score	0.9375

Recall is defined as the number of overlapping regions between the segmentation region and target region divided by the number of segmentation regions, numerically equivalent to the sensitivity. Precision is defined as the number of overlapping regions between the segmentation region and target region divided by the number of target regions. The F-1 score is the harmonic mean of precision and recall. In validation set 2, the recall of this system was 95.43%, and the precision was 92.12%.

### The DeFrame System Test Results

The DeFrame system was tested using validation set 3 for 198 videos. The results show that the DeFrame system achieves a recall of 100% and precision of 80.80%, which suggests that the system can be used to consistently detect all polyps marked by the endoscopist with a relatively low false-positive rate. The test results for the DeFrame system are shown in Table 5.

**TABLE 5:** Test results of the DeFrame system.

	Dataset 3 (video)
Recall, *n* (%)	100
Precision, *n* (%)	80.8
F-1 score	0.8938

### Speed Analysis of Colorectal Polyp Identification in the DeFrame System

**FIGURE 4:**
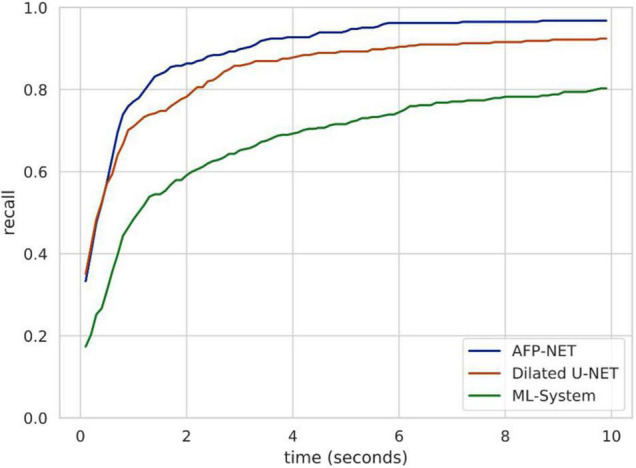
Speed analysis of polyp identification in a DeFrame system from a full-length video perspective. Recall is a function of time for different models to find a polyp. Algorithm B (based on AFP-NET) can be used to detect more than 84% of polyps within the first 2 s when they appear in the view field, while the entire system can be used to detect more than 80% of polyps within the first 10 s.

Endoscopists should detect polyps as soon as possible when they appear in the field of view during endoscopy due to many factors in clinical practice. For this purpose, we used validation set 3 to evaluate the polyp detection speed of our system. Algorithm B can be used to detect more than 84.62% of polyps within the first 2 s when they appear in the view field, while the entire system can detect more than 80.38% of polyps within the first 10 s (Figure 4). The system was tested to achieve real-time detection at approximately 23 frames per second to meet clinical practice needs.

### Analysis of the Number of False-Positives Generated

**FIGURE 5:**
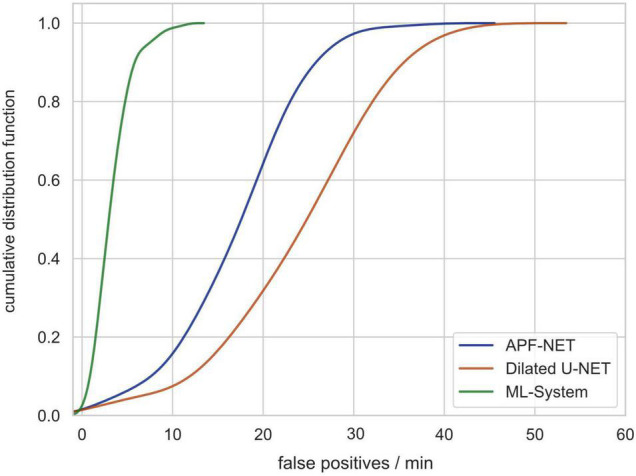
The cumulative distribution function (CDF) of the number of false-positives generated per minute per video showing that the system can avoid most false-positives.

Previous DL-based polyp detection systems are not applicable in clinical practice partially because computer-aided diagnosis (CAD) systems can produce many false-positive results. For this reason, we used video clips without polyps from validation set 3 to test the number of false-positives generated by our system (video length varies from less than 5 min to more than 30 min). As shown in Figure 5, the cumulative distribution function (CDF) of the number of false-positives generated per minute per video shows that the system can avoid most false-positives.

## Discussion

As the gold standard for CRC screening, colonoscopy has made a remarkable contribution to reducing CRC-related mortality due to its ability to directly intervene in suspected malignant polyps during colonoscopy. However, as previously mentioned, approximately one-quarter of polyps are still missed in clinical practice today. In addition, the long training time required and high entry barrier for the procedure has resulted in a severe shortage and heavy workload for endoscopists. Recently, with the development of artificial intelligence, the emergence of endoscopic CAD systems has helped in making clinical decisions and, to a certain extent, reduced human bias during colonoscopy, thus reducing the polyp miss rate (27). In this study, we constructed a DeFrame polyp detection system (DeFrame), which can detect polyps of various morphologies and locations in colonoscopy videos generated during clinical practice with good working performance.

Most polyp detection algorithms used in previous studies have been developed based on public datasets, and the sample sizes of the training and validation sets have been relatively small., e.g., in two widely used public datasets (CVC-Clinic DB and ETIS-Larib polyp DB), the number of polyps was 33 and 111, respectively. In addition, polyps in images obtained from open-source databases are generally obvious to detect with a clean background (23, 24). However, during clinical colonoscopy, most of the images are blurred with various degrees of artifacts (e.g., liquid, food residue, bubbles, reflections, mirrors, etc.), so the detection performance of these algorithms is significantly decreased when applied to clinical practice. In this study, we built a polyp image database with images extracted from endoscopic video clips and a video database of full-length videos. To the best of our knowledge, the size of both databases we used is larger than any other existing database, which ensures the accuracy of the system detection performance to some extent.

Most previous studies have been based on the use of a single algorithm, so combining sensitivity, specificity, and real-time recognition has been difficult, which has resulted in a significant proportion of false-positives (17, 18, 21, 28). Consistent with this, in our previous study, we developed two convolutional neural network (CNN)-based polyp detection models. Additionally, we found that the false-positive rates of the two models are different. Therefore, in this study, we integrated the fuzzy recognition algorithm with two different polyp detection algorithms. We found that a slight decrease in sensitivity can significantly reduce the number of false-positives and increase specificity after integration. The number of images and videos we used for this study is sufficient to produce statistically meaningful results. To the best of our knowledge, the polyp detection system (DeFrame) we have built is the first to integrate multiple deep learning-based algorithms.

As mentioned above, our polyp detection module can be subdivided into two different polyp detection algorithms and the system itself. Our DeFrame system works better when trained and tested on the same two public databases than other current polyp detection algorithms.

The traditional measurement of specificity and sensitivity does reflect the system’s performance, but there are certain flaws. The reasons for this are as follows: in clinical practice, multiple polyps can exist within a certain field of view (in a single image), and using traditional measures alone (image as a unit) can lead to one missing other polyps in the same image, resulting in an inaccurate assessment of the system’s performance. Therefore, in this study, we evaluated the system’s performance at both the image and polyp levels to better understand the system’s role in clinical care.

Additionally, we must admit that this study has some limitations. Since the DeFrame system was only trained, validated, and tested with colon images and videos, further investigation is needed to determine whether it can effectively detect polyps in areas other than the colon. In addition, we used the original video recordings to simulate clinical practice, but the clinical setting is variable, and we need prospective studies to evaluate how well the system performs in clinical practice. Finally, the endoscopic devices produced by different manufacturers vary in terms of light source or resolution, so external validation is needed to assess the system’s adaptability. A possible next step would be to incorporate AI pathology techniques to assist in predicting polyp pathology outcomes to differentiate benign and malignant polyps under endoscopy (29, 30).

## Conclusion

We developed a fast, accurate and reliable DeFrame system for detecting polyps, which, to some extent, is feasible for use in routine clinical practice. However, further investigation is needed to determine whether the system can improve the ADR in clinical practice, subsequently reducing the incidence of CRC and CRC-related mortality.

## Data Availability Statement

The original contributions presented in the study are included in the article/supplementary material, further inquiries can be directed to the corresponding authors.

## Ethics Statement

This study is in accordance with the Helsinki Declaration and has been approved by the Ethics Committee of Xiangya Hospital of Central South University (No. 201812543). Written informed consent was not required because the participants would not be identified from the colonoscopy videos.

## Author Contributions

XL, YC, and BL designed the experiments. SC, SL, DW, and XS collected the video recordings data and analyzed the data. SC, SL, JY, and YHC assembles the collected data. SL, YHC, and YC wrote and revised the manuscript. All authors read and approved the final manuscript.

## Conflict of Interest

YT was employed by the company HighWise Medical Technology Co., Ltd. The remaining authors declare that the research was conducted in the absence of any commercial or financial relationships that could be construed as a potential conflict of interest.

## Publisher’s Note

All claims expressed in this article are solely those of the authors and do not necessarily represent those of their affiliated organizations, or those of the publisher, the editors and the reviewers. Any product that may be evaluated in this article, or claim that may be made by its manufacturer, is not guaranteed or endorsed by the publisher.
